# A Patient With Three Types of Hematologic Disorders and Hemophagocytic Lymphohistiocytosis Triggered by SARS-CoV-2: A Case Report

**DOI:** 10.7759/cureus.87754

**Published:** 2025-07-11

**Authors:** Yajing Zhao, Jianjian Zhang, Jianling Qiao, Chuanfang Liu, Xinguang Liu

**Affiliations:** 1 Department of Hematology, Qilu Hospital, Cheeloo College of Medicine, Shandong University, Jinan, CHN; 2 Department of Internal Medicine, Jinan Second People's Hospital, Jinan, CHN; 3 Department of Hematology, Jinan Integrated Traditional Chinese and Western Medicine Hospital, Jinan, CHN

**Keywords:** hemophagocytic lymphohistiocytosis, immunosuppression, lymphoma, sars-cov-2, second primary malignancy

## Abstract

Second primary malignancy is a serious late complication following anti-cancer treatments including chemotherapies, immune therapies, and radiotherapies. Prolonged use of rituximab-containing immunochemotherapies in patients with non-Hodgkin lymphoma can impair immune surveillance, potentially increasing the risk of severe infections and second primary malignancies. Here, we report the case of a patient initially diagnosed with follicular lymphoma, who then developed mantle cell lymphoma 11 years later. After several rounds of rituximab-containing therapies, the patient had impaired immunity and experienced severe SARS-CoV-2 pneumonia and hyperleukocytosis, which induced the hyperinflammatory responses, resulting in hemophagocytic lymphohistiocytosis (HLH). Early intervention halted the progression of HLH; however, leukocytosis and thrombocytosis recurred, eventually leading to the diagnosis of a third hematologic malignancy - chronic myeloid leukemia. Treatment with flumatinib improved the patient’s condition. This case underscores the importance of monitoring immune depression and subsequent malignancies as more patients achieve long-term survival with hematologic cancers due to evolving therapeutic advances. Early recognition and adequate supportive therapies are crucial, especially in managing elderly patients with malignancies.

## Introduction

The rapid advancements in immunotherapies and targeted therapies in recent years have greatly extended the survival of patients with hematologic malignancies. However, these treatments come with an increasing risk of long-term complications, including second primary malignancies. Studies showed that 2-17% of cancer patients developed more than two cancers in their lifetime [1]. Factors contributing to second primary malignancies in patients with diffuse large B-cell lymphoma (DLBCL) included disease stage, radiotherapy, rituximab use, and age. Lenalidomide use was reported to be associated with second primary malignancies in myeloma trials but not in lymphoma, chronic lymphocytic leukemia, or myelodysplastic syndrome trials [2].

Rituximab, a monoclonal antibody targeting CD20, induces prolonged B-cell depletion, which impairs both humoral immunity and antigen presentation, thereby weakening immune surveillance against infections and emerging malignant clones [3,4]. Patients with lymphoma have impaired immunity and are at a higher risk of SARS-CoV-2-associated morbidity and mortality, often with delayed virus clearance [5]. It has been reported that some patients, especially those under immune suppression, experienced cytokine storm during severe SARS-CoV-2 infection, which triggered the development of secondary hemophagocytic lymphohistiocytosis (HLH), a rare yet potentially fatal complication caused by excessive immune activation following severe infection, malignancy, or autoimmunity [6-8]. This report presented the case of a patient with three types of hematologic malignancies, initially follicular lymphoma (FL), followed by mantle cell lymphoma (MCL), and subsequently chronic myeloid leukemia (CML) complicated by severe SARS-CoV-2 pneumonia and HLH during the COVID-19 pandemic.

## Case presentation

**Figure 1:**
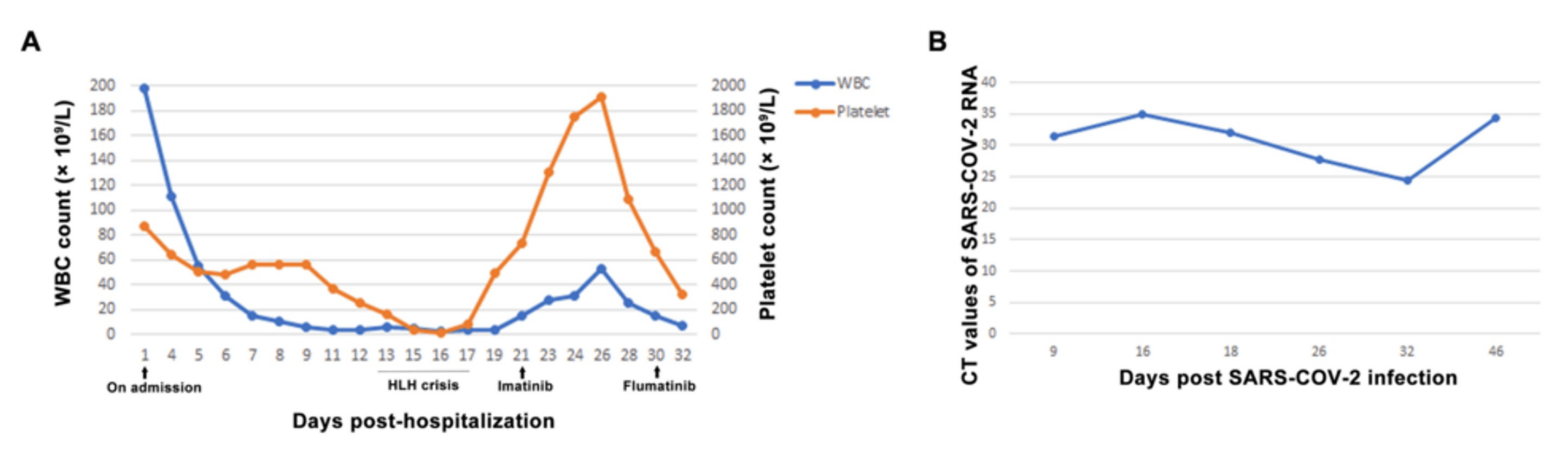
Patient's blood cell counts and viral loads (A) The trajectory of WBC and platelet counts during patient's hospitalization. (B) The CT values of the SARS-CoV-2 nucleic acid testing. WBC, white blood cell.

In September 2023, a patient in her 70s with a 14-year history of non-Hodgkin lymphoma (NHL), a 10-year history of asthma, and an 8-year history of cerebral infarction and hypertension was admitted to Qilu Hospital, Shandong University, China. The patient presented with a seven-day history of cough and fever. The patient got primary SARS-CoV-2 infection and had received a cycle of Paxlovid (nirmatrelvir 300 mg and ritonavir 100 mg, twice daily for five days). Despite this, her symptoms worsened with shortness of breath and lower extremity edema. Blood routine tests showed severe leukocytosis, thrombocytosis, and anemia (Table 1, Figure 1A).

**Table 1:** Patient’s laboratory tests Time points: on admission (September 4, 2023), during HLH crisis (September 16, 2023), post-HLH recovery (September 24, 2023), post-CML treatment (May 24, 2025). ALT, alanine aminotransferase; APTT, activated partial thromboplastin time; AST, aspartate aminotransferase; BUN, blood urea nitrogen; CK-MB, creatine kinase-MB isoenzyme; CML, chronic myeloid leukemia; Cr, creatinine; cTnI, cardiac troponin I; DBIL, direct bilirubin; Fib, fibrinogen; HLH, hemophagocytic lymphohistiocytosis; IBIL, indirect bilirubin; NT-proBNP, N-terminal pro-B-type natriuretic peptide; PT, prothrombin time; TBIL, total bilirubin; TG, triglycerides; WBC, white blood cell

Laboratory test	On admission	During HLH crisis	Post-HLH recovery	Post-CML treatment	Reference range
WBC count	198.38 × 10⁹/L	2.15 × 10⁹/L	31.35 × 10⁹/L	7.97 × 10⁹/L	4.0–10.0 × 10⁹/L
Hemoglobin	86 g/L	62 g/L	76 g/L	87 g/L	115–150 g/L
Platelet count	866 × 10⁹/L	15 × 10⁹/L	1755×10⁹/L	381 × 10⁹/L	125–350 × 10⁹/L
ALT	42 IU/L	74 IU/L	43 IU/L	8 IU/L	9–50 IU/L
AST	50 IU/L	124 IU/L	80 IU/L	10 IU/L	15–40 IU/L
TBIL	13 μmol/L	63.9 μmol/L	22.3 μmol/L	13.3 μmol/L	5.0–21.0 μmol/L
IBIL	4 μmol/L	42.1 μmol/L	5.9 μmol/L	11.5 μmol/L	2.0–15.0 μmol/L
DBIL	9 μmol/L	21.8 μmol/L	16.4 μmol/L	1.8 μmol/L	<6.0 μmol/L
PT	18.8 s	15.6 s	14.8 s	16.3 s	8.8–13.8 s
APTT	49.8 s	36.4 s	37 s	29.1 s	26–42 s
Fib	13.58 g/L	2.04 g/L	2.02 g/L	6.44 g/L	2.0–4.0 g/L
D-dimer	2.9 μg/mL	1.37 μg/mL	2.88 μg/mL	0.02 μg/mL	< 0.5 μg/mL
TG	-	0.75 mmol/L	-	-	0.3–1.7 mmol/L
Ferritin	-	3762 ng/mL	1110 ng/mL	-	30–400 ng/mL
Cr	219 μmol/L	58 μmol/L	43 μmol/L	96 μmol/L	53–97 μmol/L
BUN	12.8 mmol/L	11.6 mmol/L	6.1 mmol/L	8.9 mmol/L	2.3–7.8 mmol/L
Na^+^	131 mmol/L	129 mmol/L	133 mmol/L	133 mmol/L	137–147 mmol/L
K^+^	3.23 mmol/L	3.94 mmol/L	4.9 mmol/L	5.37 mmol/L	3.5–5.3 mmol/L
NT-proBNP	35000 pg/mL	9829 pg/mL	3155 pg/mL	1176 pg/mL	-
CK-MB	9.7 ng/mL	-	-	-	5–­­7 ng/mL
cTnI	256.01 ng/L	58.24 ng/L	-	-	< 17.5 ng/L
Lactate	4.8 mmol/L	6 mmol/L	1.4 mmol/L	-	0.5–2.2 mmol/L
FiO_2_	165	248	342	-	-

Follicular lymphoma since 2009

Fourteen years ago, the patient had submaxillary lymph node enlargement and was diagnosed with FL (stage IIIA) in Peking Union Medical College Hospital, via the lymph node biopsy. The histopathologic examination confirmed diffuse follicular central B-cell lymphoma, with immunohistochemistry staining of CD21 (+), CD3 (+), CD20 (++), CD10 (-), Ki67 (3%), PAX-5 (++), BCL-2 (+), and Bcl-6 (-). The patient received six cycles of R-CVP regimen (rituximab 600 mg, d1; cyclophosphamide 1 g, d1; vindesine 4 mg, d1; and dexamethasone 15 mg, d1-d5), followed by six cycles of rituximab monotherapy (600 mg monthly). Five years later, the patient got enlargement of cervical and inguinal lymph nodes, but she refused further biopsy and assessment of lymphoma status. The patient then underwent two cycles of R-ESHAP regimen (rituximab 600 mg, d1; etoposide 60 mg q12h, d1-d3; cisplatin 40 mg, d1-d4; cytarabine 3 g, d5; and methylprednisolone 500 mg, d1-d5), followed by more than 10 cycles of rituximab (600 mg) monotherapy intermittently.

Mantle cell lymphoma diagnosed in 2021

**Figure 2:**
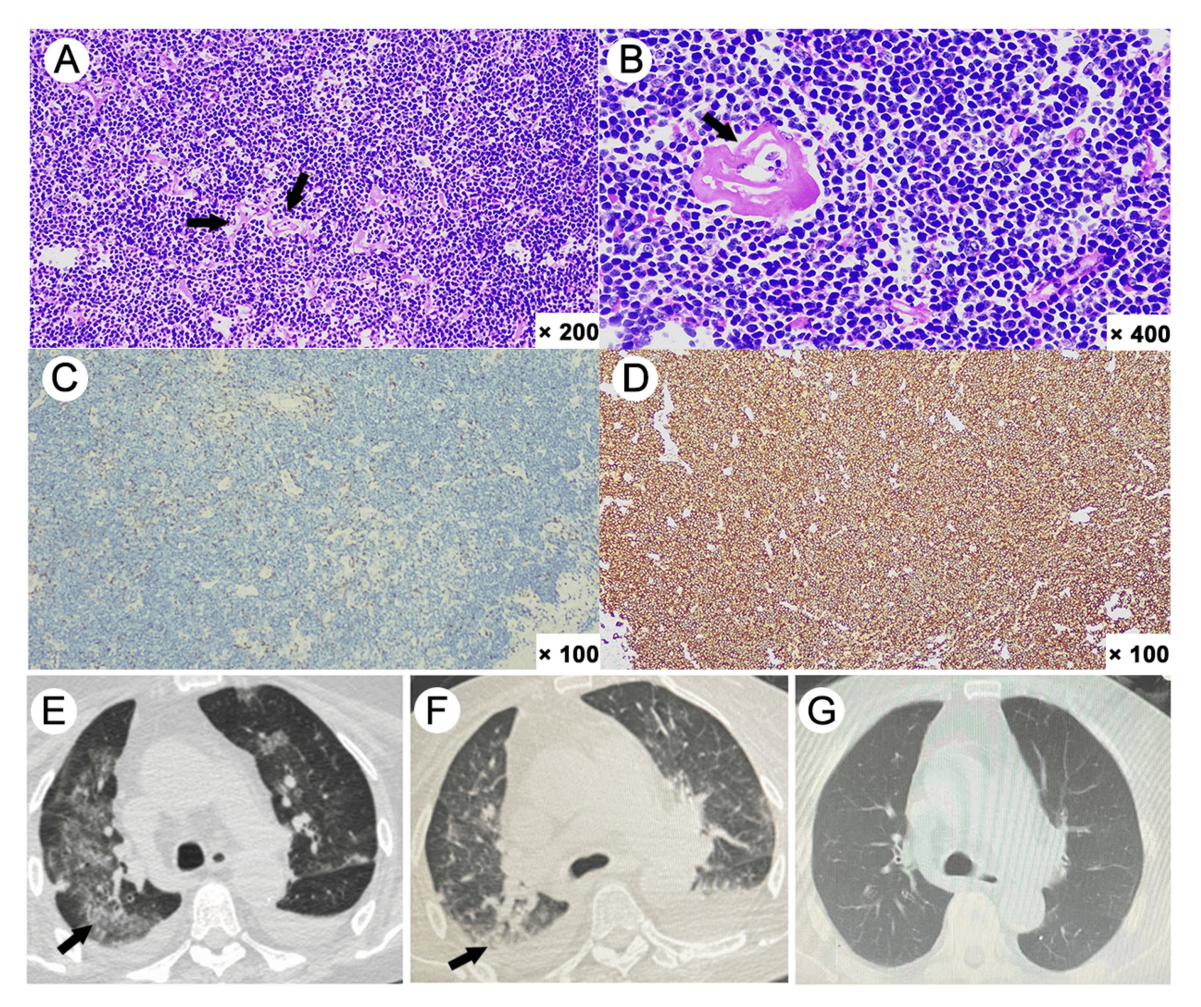
Histopathological features of the inguinal lymph node biopsy and chest CT scans (A) Hyalinosis of interstitial fibrous tissue (H&E stain, ×200). (B) Hyalinosis of the blood vessel wall (H&E stain, ×400). (C) CD3 staining was positive for the residual T cells (× 100). (D) Lymphoma cells were positive for CD20 (×100). (E) Bilateral pneumonia with pleural effusion on admission. (F) Improvement of pneumonia and absorption of pleural effusion 12 days after treatments. (G) Recovery from the severe SARS-COV-2 pneumonia at the six-month follow-up.

**Figure 3:**
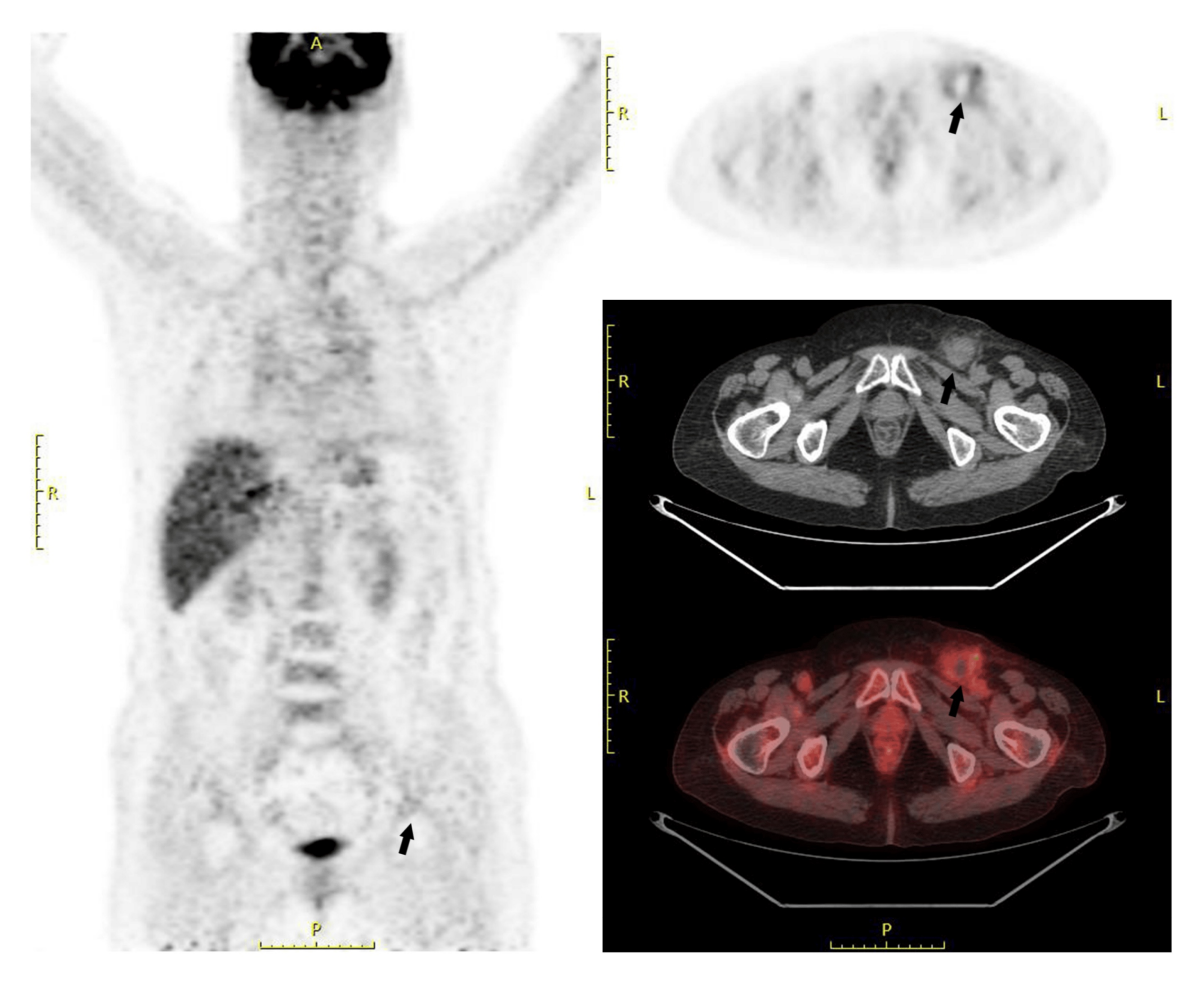
Images of PET-CT scan PET-CT scan showed limited fluorine-18 fluorodeoxyglucose (18F-FDG) uptake in the left inguinal region after the inguinal lymph node biopsy.

In February 2021, due to inguinal lymph node enlargement, fatigue, and abdominal distension, the patient was admitted to Qilu Hospital, Shandong University, and underwent inguinal lymph node biopsy. Immunohistochemistry staining showed CD20 (+), CD79a (+), CyclinD1 (+), CD3 (residual T cells +), CD5 (residual T cells +), CD23 (residual FDC +), CD21 (residual FDC+), LEF1 (T cells +), CD10 (-), Bcl-6 (-), CD30 (scattered +), Bcl-2 (+), PAX-5 (+), CD43 (+), SOX11 (+), c-Myc (-), MUM-1 (-), P53 (-), CD38 (-), CD138 (-), Ki-67 (20%), and EBER (-) (Figures 2A-2D). The patient was then diagnosed with MCL (stage IA) according to the histopathological examination. PET-CT scan showed limited inguinal lymphadenopathy (Figure 3). The patient then received three cycles of rituximab (600 mg monthly) and lenalidomide (25 mg d1-d10), later switching to orelabrutinib (150 mg daily) for three days before the cerebral infarction occurred (Table 2). The patient continued irregular rituximab monotherapy, with the latest dose received seven months before her SARS-CoV-2 infection. The patient received more than 30 cycles of rituximab (600 mg/cycle) starting from her diagnosis of FL (Table 2).

**Table 2:** Timeline of NHL therapy R-CVP regimen: rituximab (375 mg/m^2^, d1), cyclophosphamide (750 mg/m^2^, d1), vindesine (4 mg, d1), and dexamethasone (15 mg, d1-d5); R-ESHAP regimen: rituximab (375 mg/m^2^, d1), etoposide (40 mg/m^2^ q12h, d1-d3), cisplatin (25 mg/m^2^/day, d1-d4), cytarabine (2 g/m^2^, d5), and methylprednisolone (500 mg d1-d5). NHL, non-Hodgkin lymphoma

Diagnosis	Time period	Therapies
Follicular lymphoma	February 2009 to March 2010	R-CVP regimen × six cycles; rituximab monotherapy × two cycles
	May 2010 to March 2011	Rituximab monotherapy × four cycles
Cervical and inguinal lymph node enlargements	April 2014 to December 2014	R-ESHAP regimen × two cycles; rituximab monotherapy × two cycles
Inguinal lymph node enlargement	June 2016 to October 2016	Rituximab monotherapy × three cycles
	November 2018 to May 2019	Rituximab monotherapy × four cycles
	March 2020 to December 2020	Rituximab monotherapy × five cycles
Mantle cell lymphoma	February 2021 to May 2021	Rituximab and lenalidomide × three cycles
	November 2021	Orelabrutinib × three days

CML diagnosed in 2023 during SARS-CoV-2 infection

Upon admission for SARS-CoV-2 pneumonia, the patient exhibited fever, dyspnea, and tachycardia. Blood tests and CT scan confirmed severe pneumonia with co-infection (Figures 2E, 2F), and multi-organ dysfunction (MODS), including type 1 respiratory failure, heart failure, acute kidney injury, liver dysfunction, coagulation disorders, and electrolyte disturbances (Table 1). The patient was treated with hydroxyurea (1 g, twice daily) for hyperleukocytosis, molnupiravir (0.8g, every 12 hours) for SARS-CoV-2 considering the kidney injury, and a combination of meropenem (1 g, every 8 hours), tigecycline (100 mg, every 12 hours), and caspofungin (50 mg, daily) for bacterial infection and fungal prophylaxis. The patient received timely supportive treatments, including oxygen supplementation, circulatory support with alprostadil and recombinant human atrial natriuretic peptide (rhANP), correction of acid-base and electrolyte imbalance, cardioprotective therapy with creatine phosphate, coenzyme Q10, and statins, and hepatoprotective therapies. The patient’s condition initially stabilized, with improvement of temperature and dyspnea. Bone marrow examination revealed reactive granulocytic changes, with a neutrophil alkaline phosphatase (NAP) positive rate of 95% and a total NAP score of 320. Tissue cells and phagocytes were readily observed. Flow cytometry analysis did not show abnormal cells phenotypically. Of note, fusion gene screening revealed positive of BCR::ABL1 p210 (International Scale [IS] 121.9%), confirmed by karyotype analysis (46, XX, t(9;22)(q34.1;q11.2)), indicative of CML. Gene sequencing identified ASXL1 (47.56%) and BCOR (51.35%) mutations. The patient had no splenomegaly, with the Sokal score of 1.07. CT scans showed no lymph node enlargement, and thus her NHL was at a stable stage.

HLH triggered by SARS-CoV-2 deterioration

Before initiating tyrosine kinase inhibitor (TKI) therapy, the patient’s blood cells dropped rapidly even though hydroxyurea had already been stopped (Table 1, Figure 1A). The temperature increased again, with elevated ferritin levels. Liver function was worsened, while the triglyceride level and blood coagulation tests, including fibrinogen, were in a normal range (Table 1). Although evaluation of soluble IL-2R and NK cell activity was unavailable in our center, the patient was diagnosed with an HLH crisis based on an HScore of 174 [9]. Dexamethasone at 15 mg/day (10 mg/m2) and intravenous immunoglobin (IVIg) at 20 g/day for five days were promptly administered upon suspicious diagnosis of HLH. The patient’s blood cells dropped to a nadir and then gradually recovered with leukocytosis and thrombocytosis (Table 1, Figure 1A). Blood cytology was performed again, which showed elevated WBC count with early, middle, and late granulocytes accounting for 26%, with occasionally observed basophils and increased ratio of monocytes. Although initially intolerant to imatinib (400 mg daily, later reduced to 300 mg due to severe vomiting), the patient was successfully switched to flumatinib (600 mg daily).

SARS-CoV-2 nucleic acid remained positive after 25 days of molnupiravir administration (Figure 1B). The patient was in a severe immunosuppressive state with B cell count at 0/μL, T cells at 423/µL, and NK cells at 7/µL. The patient completed additional two cycles of Paxlovid with intermittent plasma transfusions. After 46 days, the patient finally recovered from the pneumonia and continued the targeted therapy for CML (Figure 1B). Follow-up in March 2024 showed normal chest CT scans (Figure 2G), and recent blood tests in May 2025 showed stable health conditions with BCR::ABL1 p210 fusion gene decreased to 0.0149% on the IS (Table 1). 

The diagnostic criteria related to this case are listed in Table 3.

**Table 3:** Diagnostic criteria NAP, neutrophil alkaline phosphatase

Disease	Key features	Details
Follicular lymphoma [10]	Histopathology	Nodular/follicular growth with centrocytes and centroblasts
	Immunophenotype	Positive for monoclonal immunoglobulin light chain, CD19, CD20, CD10, BCL2, and BCL6; negative for CD5 and CD23
	Cytogenetics	t(14;18)(q32;q21); IGH::BCL2 fusion
	Staging	Ann Arbor system
Mantle cell lymphoma [11]	Histopathology	Monotonous small-to-medium lymphoid cells with irregular nuclei
	Immunophenotype	Positive for CD20, CD5, cyclin D1, SOX11
	Cytogenetics	t(11;14)(q13;q32) causing cyclin D1 overexpression
	Staging	Ann Arbor system
Chronic myeloid leukemia [12]	Peripheral blood	Marked leukocytosis, left-shift granulocytosis, thrombocytosis, basophilia
	Bone marrow	Hypercellular with granulocytic hyperplasia
	Cytogenetics/molecular	t(9;22)(q34;q11) (Philadelphia chromosome); BCR::ABL1 fusion
	NAP score	Low in chronic myeloid leukemia (can be elevated during infection)
Hemophagocytic lymphohistiocytosis	Diagnosis (hemophagocytic lymphohistiocytosis 2004): ≥5 of 8 [13]	1. fever; 2. splenomegaly; 3. cytopenias (≥2 lineages); 4. hypertriglyceridemia and/or hypofibrinogenemia; 5. hemophagocytosis (no malignancy); 6. low/absent NK cell activity; 7. ferritin ≥ 500 ng/mL; 8. elevated sCD25 (IL-2R)
	Alternative [9]	HScore ≥ 169 with a sensitivity of 93% and specificity of 86%

## Discussion

NHL is the most common hematologic malignancy, which is etiologically and clinically heterogeneous [14]. Patients with NHL are at a higher risk of second primary malignancies compared to the general population, largely due to repeated exposure to genotoxic therapeutic agents and prolonged primary and secondary immunodeficiencies [3]. This report highlights the case of a patient with a long-standing history of NHL, initially diagnosed as FL. Over the course of treatment, which included repeated cycles of anti-CD20 therapies and cytotoxic agents, the patient developed MCL and subsequently, during an acute SARS-Cov-2 infection, was diagnosed with CML.

FL is an indolent B-cell lymphoproliferative disorder, accounting for approximately 35% of all NHLs and 70% of indolent lymphomas [10]. Histologic transformation of FL into more aggressive forms, most commonly DLBCL, occurs in approximately 10% to 70% of cases over time, with an estimated annual risk of 2%. Other types of transformation, including Hodgkin-like disease, plasmablastic lymphomas, lymphoblastic lymphomas, and histiocytic sarcomas/dendritic cell neoplasms, are rarely reported [15]. In this case, 11 years after the initial diagnosis of FL, biopsy of an enlarged inguinal lymph node revealed MCL. Given MCL’s origin from naïve B cells in the mantle zone and its distinct immunophenotypic and molecular profile, the MCL is not considered a transformation but rather a composite lymphoma. Composite lymphomas comprising both FL and MCL are exceedingly rare. Notably, the MCL component in such cases often exhibits indolent behavior and lacks features of aggressive transformation [16]. Consistent with these observations, the patient’s MCL was localized on PET-CT and remained stable even after treatment discontinuation.

During the long period of NHL treatments, the patient received more than 30 cycles of rituximab, which significantly impaired her immunity. At hospital admission for severe SARS-CoV-2 infection, our patient’s B cell counts were undetectable (0/μL), with remarkably reduced levels of T cells and NK cells. Apart from humoral immunity, B cells contribute to anti-tumor immunity by presenting tumor-associated antigens and producing immunostimulatory cytokines [17]. Loss of B-cell-mediated immune surveillance may impair early detection and elimination of emerging malignant clones, including those harboring mutations such as ASXL1 or BCOR, which were identified in this patient. Prolonged exposure to rituximab eliminates CD20⁺ B cells and also impairs the homeostasis of T cells and NK cells, thus predisposing the patients to both severe infections and secondary malignancies [3,4]. This was her first SARS-CoV-2 infection during the COVID-19 pandemic. Since the patient was in an extremely immunodeficient state, the patient got severe pneumonia, leading to MODS in a short time. Immunocompromised individuals, especially those with hematologic malignancies, are at a high risk of SARS-CoV-2-associated morbidity and mortality, largely due to immunologic deficits that hinder virus clearance [5]. The patient did not receive COVID-19 vaccination before her infection, which might also account for her rapid deterioration upon SARS-CoV-2 infection. It is still controversial whether COVID-19 vaccination could benefit in patients with hematological malignancies. It has been reported that patients with hematologic malignancies presented with impaired immune response to COVID-19 vaccination, especially in patients with lymphomas, who displayed a largely impaired seroconversion rate of around 55% after two doses of vaccination and 33% after one dose [18,19]. Interestingly, patients with multiple sclerosis receiving anti-CD20 therapy demonstrated active T-cell responses to COVID-19 vaccination, underscoring the protective role of vaccination in individuals with B-cell deficiency [20]. Nevertheless, COVID-19 vaccines are generally safe for patients with hematological malignancies.

In this patient, SARS-CoV-2 persisted for over a month despite multiple rounds of anti-viral treatments and plasma transfusions, eventually culminating in HLH. SARS-CoV-2 could trigger cytokine storm especially in severely ill patients, which might be an igniter of HLH [6]. As the SARS-COV-2 pandemic progresses, increased numbers of patients are reporting rare but severe complications such as HLH [8]. HLH is a life-threatening disease and usually progresses rapidly. Early recognition and therapies with IVIg and corticosteroids are required upon suspicion of HLH to achieve successful outcomes [21]. Although the patient had two types of NHL, both lymphomas were relatively stable and insufficient to trigger HLH immune reactions by themselves. HScore is a useful tool for suspected HLH in SARS-COV-2 patients [22,23]. Higher HScores (>98) in suspected patients with SARS-COV-2 infection are associated with a more severe disease progression and higher mortality rate [23]. The patient had an HScore of 174, which possessed a 93% sensitivity and 86% specificity for HLH [9].

Second primary malignancies were increasingly recognized as a consequence of longer survival time in patients with malignancies. CML is rarely reported as a therapy-related disorder secondary to NHL [24]. This patient developed two different second primary malignancies, MCL and CML, over the long course of NHL treatments, which was rarely seen in clinical practice. MCL was diagnosed 11 years after initial FL treatments, and hyperleukocytosis noted during a SARS-COV-2 infection promoted further investigation, leading to the CML diagnosis. Hyperleukocytosis was initially suspected to result either from the progression of NHL or from a hyper-inflammatory reaction due to the severe infection. However, the degree of leukocytosis, the presence of thrombocytosis, and the repeated previous immunochemotherapies raised suspicion for an underlying clonal process - acute leukemia following lymphoma progression or hyperinflammatory reaction rarely presents with distinct thrombocytosis. We conducted bone marrow aspiration and cytogenetic testing, which ultimately confirmed the diagnosis of chronic-phase CML via identification of the Philadelphia chromosome and BCR::ABL1 fusion gene [12].

Besides the BCR::ABL1 fusion gene, next-generation sequencing revealed concurrent mutations in ASXL1 and BCOR, both of which are epigenetic regulators associated with myeloid neoplasms. ASXL1 is the most frequently mutated gene beyond BCR::ABL1 in CML, reported in approximately 9%-14% of cases, particularly in accelerated and blast phases. ASXL1 mutations are associated with inferior responses to TKIs, increased risk of transformation to myelodysplastic syndromes or acute leukemia, and impaired survival [25-27]. BCOR mutations, though less prevalent, have also been noted in the blast phase of CML and are associated with unfavorable treatment outcomes [28]. The co-occurrence of ASXL1 and BCOR mutations in the patient indicates genetic instability that may contribute to disease progression and resistance to TKI regimens, which warrants close monitoring. Although imatinib would be an appropriate choice for this patient, it was not tolerated due to severe gastrointestinal side effects. Dasatinib and nilotinib were not selected because of the patient’s pre-existing pleural effusion and heart failure, which are relative contraindications [29]. In the event that flumatinib is unavailable, other next-generation TKIs may serve as viable alternatives.

Repeated rituximab uses significantly compromised the patient’s immunity, resulting in persistent SARS-COV-2 infection and related severe pneumonia and HLH. After several rounds of anti-viral and antibiotic treatments, the patient recovered from pneumonia, and the blood cell counts normalized following TKI treatments. Despite the complexities in managing the tumors and severe SARS-COV-2 pneumonia, the patient has returned to normal life. Among the three hematologic malignancies, FL and MCL were stable, while CML required ongoing treatment.

## Conclusions

This case highlights the rare and complex clinical course of a patient who sequentially developed three distinct hematologic malignancies - FL, MCL, and CML - culminating in a life-threatening episode of HLH triggered by severe SARS-CoV-2 infection. As more patients achieve long-term survival with hematologic cancers due to evolving therapeutic advances, this case highlights the critical need for vigilant monitoring of immunosuppression and second primary malignancies, especially in patients under extensive exposure to immunochemotherapies such as rituximab. Hyperleukocytosis under severe SARS-CoV-2 infection with cytokine storm complicated the diagnosis of CML, emphasizing the need for cytogenetic and molecular screening for differential diagnosis. Prolonged immunosuppression from hematologic malignancies and their treatments can lead to impaired viral clearance and, rarely, may cause secondary HLH, for which timely recognition and appropriate supportive interventions remain essential. Interdisciplinary collaboration is critical in managing the elderly and immunocompromised individuals with overlapping oncologic and infectious pathologies. Early identification of immune dysfunction, long-term follow-up for secondary malignancies, and prompt, targeted interventions remain essential for improving outcomes in this growing patient population.
